# Blood Astrocyte Biomarkers in Alzheimer Disease: A Systematic Review and Meta-Analysis

**DOI:** 10.1212/WNL.0000000000209537

**Published:** 2024-07-10

**Authors:** Sarah Holper, Paula Loveland, Leonid Churilov, Dominic Italiano, Rosie Watson, Nawaf Yassi

**Affiliations:** From the Population Health and Immunity Division (S.H., P.L., R.W., N.Y.), The Walter and Eliza Hall Institute of Medical Research; Department of Medicine (S.H., P.L., L.C., D.I., R.W., N.Y.), The Royal Melbourne Hospital, and Department of Neurology (N.Y.), Melbourne Brain Centre at The Royal Melbourne Hospital, University of Melbourne, Parkville, Australia.

## Abstract

**Background and Objectives:**

Neuroinflammation, particularly early astrocyte reactivity, is a significant driver of Alzheimer disease (AD) pathogenesis. It is unclear how the levels of astrocyte biomarkers change in patients across the AD continuum and which best reflect AD-related change. We performed a systematic review and meta-analysis of 3 blood astrocyte biomarkers (glial fibrillary acidic protein [GFAP], chitinase-3-like protein 1 [YKL-40], and S100B) in patients clinically diagnosed with AD.

**Methods:**

MEDLINE and Web of Science were searched on March 23, 2023, without restrictions on language, time, or study design, for studies reporting blood levels of the astrocyte biomarkers GFAP, YKL-40, or S100B in patients on the AD continuum (including those with mild cognitive impairment [MCI] and dementia) and a cognitively unimpaired (CU) control population. AD diagnosis was based on established diagnostic criteria and/or comprehensive multidisciplinary clinical consensus. Studies reporting indirect biomarker measures (e.g., levels of biomarker autoantibodies) were excluded. Risk of bias assessment was performed using the revised Quality Assessment of Diagnostic Accuracy Studies tool. Pooled effect sizes were determined using the Hedge *g* method with a random-effects model. The review was prospectively registered on PROSPERO (registration number CRD42023458305).

**Results:**

The search identified 1,186 studies; 36 met inclusion criteria (AD continuum n = 3,366, CU n = 4,115). No study was assessed to have a high risk of bias. Compared with CU individuals, patients on the AD continuum had higher GFAP and YKL-40 levels (GFAP effect size 1.15, 95% CI 0.94–1.36, *p* < 0.0001; YKL-40 effect size 0.38, 95% CI 0.28–0.49, *p* < 0.0001). Both biomarkers were elevated in more advanced clinical stages of the disease (i.e., in AD dementia compared with MCI due to AD: GFAP effect size 0.48, 95% CI 0.19–0.76, *p* = 0.0009; YKL-40 effect size 0.34, 95% CI 0.10–0.57, *p* = 0.0048). No significant differences in blood S100B levels were identified.

**Discussion:**

We demonstrated significant elevations in blood GFAP and YKL-40 levels in patients on the AD continuum compared with CU individuals. Furthermore, within the AD clinical spectrum, significant elevation correlated with more advanced disease stage. Our findings suggest that both biomarkers reflect AD-related pathology. Our findings are limited by the lack of cultural and linguistic diversity in the study populations meta-analyzed. Future meta-analyses using a biomarker-defined AD population are warranted.

## Introduction

Alzheimer disease (AD), considered a clinical syndrome for over a century, is undergoing a profound redefinition. In 2018, the National Institute on Aging-Alzheimer's Association (NIA-AA)'s clinical research criteria reconceptualized AD as a biologically defined entity, diagnosed in vivo by biomarker evidence of the 2 core hallmarks of the disease: β-amyloid (Aβ) proteinopathy (A) and hyperphosphorylated tau (T).^1^ Rigorous research has continued to consolidate and expand the role of AD biomarkers, particularly those that are blood-based, facilitated by advances in ultrasensitive assay techniques.

Supporting AD's transition from a clinical diagnosis to a biologically defined entity requires a robust understanding of which biomarkers accurately reflect AD-related pathology. Although the core A and T biomarkers define the disease, derangements in biomarkers reflecting concurrent pathologic processes involved in AD, but not unique to it, provide important complementary information. It is now well-established that neuroinflammation, particularly early astrocyte reactivity, is a significant driver of AD pathogenesis.^2^ Biomarkers of neuroinflammation are anticipated to adopt an increasingly prominent role as our understanding of AD-related immune responses develops.

Astrocytes, the most abundant cells in the CNS, perform vital metabolic, homeostatic, structural, and neuroprotective functions. Astrogliosis refers to the marked modifications in morphology, function, and gene expression that astrocytes undergo as a defensive response to CNS injury, including AD pathology. Proteins released into the CSF and blood by such reactive or activated astrocytes may serve as in vivo fluid biomarkers of this immune response. Pronounced alterations in the astrocyte expression of glial fibrillary acidic protein (GFAP), S100B, and chitinase-3-like protein 1 (YKL-40) have been identified in patients with AD. Clinical corollaries are emerging; for instance, it is recognized that an AD phenotype with blood GFAP elevation confers more rapid cognitive decline.^3^

Robust debate surrounds which biomarkers of neuroinflammation best reflect AD-related change. A key consideration is a biomarker's performance when measured in blood, given the ease of sample attainment compared with CSF. Rapid progress in ultrasensitive protein detection techniques, particularly single-molecule array technology, has recently enabled the development of accurate and reliable blood astrocyte biomarkers, accompanied by a surge in publications using this new technology. A 2021 meta-analysis including 2 blood astrocyte biomarkers, GFAP and S100B, found a significant difference between the AD and cognitively unimpaired (CU) groups for S100B, but not for GFAP.^4^ However, of the small number of eligible studies (5 for each biomarker), only 2 reported the direct blood biomarker level: the other 8 measured autoantibodies to the biomarker or levels in plasma astrocyte-derived exosomes. It is unclear how the heterogeneity in the species meta-analyzed may have affected on the findings.

Although this approach provides valuable information on the potential role of GFAP and S100B in AD pathogenesis and progression, a novel meta-analysis limited to techniques which measure absolute levels of the specific analytes in blood, particularly given the considerable number of recent publications reporting such results, is required in preparation for future clinical implementation. We therefore conducted a systematic review and meta-analysis of the blood levels of the astrocyte biomarkers GFAP, YKL-40, and S100B to investigate whether different stages on the AD clinical spectrum are characterized by different levels of biomarkers.

## Methods

### Standard Protocol Approvals, Registrations, and Patient Consents

We followed the Preferred Reporting Items for Systematic Reviews and Meta-analyses guidelines when completing this systematic review and meta-analysis. The review was prospectively registered on PROSPERO (registration number CRD42023458305). Obtaining patient consent was not applicable for this systematic review and meta-analysis.

### Search Strategy

MEDLINE and Web of Science were searched without any restrictions on language, time, or study design on March 23, 2023. The comprehensive search strategy is detailed in eMethods.

### Eligibility Criteria and Data Extraction

Identified studies were uploaded to Covidence, a web-based research tool to facilitate streamlined screening and data extraction for the purposes of systematic reviews. Two authors (S.H. and P.L.) independently performed title and abstract screening. Exclusion criteria included studies not pertaining to AD, reviews or editorials, studies not conducted on living humans (e.g., postmortem analyses, in vitro studies, and animal models), studies in which patients with AD had additional acute pathology (e.g., coronavirus disease 2019 infection), or studies not written in English. All studies deemed eligible by at least 1 author proceeded to full-text review.

The same 2 authors performed full-text review, blinded to the other's verdict. Inclusion required the study to be of an observational design in which levels of blood astrocyte biomarkers (1 or more of S100B, YKL-40, or GFAP) were measured in both patients with sporadic AD and a CU control population. Included studies reported direct blood levels of these biomarkers: not levels of autoantibodies to them or levels obtained from plasma astrocyte-derived exosomes. Data from both cross-sectional studies, and baseline data from longitudinal studies, were included. AD diagnosis must have been based on established diagnostic criteria and/or comprehensive multidisciplinary clinical consensus (see eMethods for criteria used). Patients must have been on the AD clinical continuum (i.e., cognitively impaired), expressed as a diagnosis of mild cognitive impairment due to AD (MCI-AD), AD dementia, or a combined cohort of both. Biomarker levels must have been presented as a summary statistic: either mean and SD or SEM, or median and interquartile range (IQR) or range. If data were presented in graphical form (e.g., funnel plots), the authors were contacted by email with a request to provide a summary statistic; lack of reply resulted in study exclusion. Where 2 studies analyzed data from the same cohort, the study with the smaller sample size was excluded (eTable 1). Discordant eligibility assessments were resolved by consensus through discussion between the same 2 authors.

### Data Collection Process

S.H. performed data extraction. Data extracted included sample size, demographics (e.g., age and female preponderance), clinical diagnosis in the study's AD cohort/s, the diagnostic criteria used, the method of biomarker quantification, and the relevant biomarker level/s. For each cohort, the clinical diagnosis on the AD continuum was recorded as either MCI-AD (i.e., patients in the MCI stage), AD dementia (i.e., patients in the dementia stage), or “AD dementia + MCI-AD” (for cohorts comprising patients with both MCI-AD and AD dementia). Where available, the area under the receiver operating curve (AUC) for the biomarker's discriminatory ability was extracted.

### Risk of Bias Assessment

Risk of bias assessment was conducted by S.H. based on the revised Quality Assessment of Diagnostic Accuracy Studies tool, designed for use in systematic reviews of diagnostic accuracy.^5^ Signaling questions pertaining to the 4 domains of patient selection, index test, reference standard, and flow and timing were adapted for application to this study. The signaling questions and bias assessments can be found in eTables 2a and 2b, respectively.

### Statistical Analysis

Studies were grouped according to the biomarker/s of interest that they measured. For each biomarker, we pooled individual study effects expressed as Hedge *g* to estimate the mean pooled effect size for CU individuals compared with AD continuum patients (i.e., a combination of all MCI-AD cohorts, AD dementia cohorts, and AD dementia + MCI-AD cohorts). Next, AD cohorts were grouped according to clinical diagnosis, and effect sizes calculated for all available comparisons (i.e., comparing MCI-AD with CU, AD dementia with CU, and MCI-AD with AD dementia; data from AD dementia + MCI-AD cohorts were only included in the initial AD continuum analysis). Finally, we pooled AUC data from studies where these were available for a biomarker of interest, to calculate a mean pooled AUC, with corresponding 95% CI.

All statistical analyses were performed using Stata version 17.0 (StataCorp, College Station, TX). For all analyses, to estimate pooled effects, we used a random-effects meta-analysis using the model of restricted maximum likelihood. An effect size between 0.2 and 0.5 was considered small; a medium effect size was between 0.5 and 0.8; and a large effect size was 0.8 or greater.^6^ Heterogeneity (the proportion of the variation in observed effects that is due to variation in true effects) was estimated using the *I*^2^ index.^7^ In cases where several AD cohorts were compared with 1 control group, the control group's sample size was divided by the number of comparisons to avoid effect size overestimation. Publication bias was assessed using visual inspection of funnel plots and the Egger regression test.

We did not exclude studies that presented biomarker levels as median-based rather than mean-based summary values. To facilitate meta-analysis of all studies for each biomarker, regardless of how data were presented, data from studies presenting median-based results underwent transformation to yield an estimated sample mean and SD. We performed transformation using 2 techniques. The first technique used a Box-Cox transformation.^8^ The second used published methods for estimating a sample mean^9^ and SD^10^ from median-based results, after assessing for skewness.^11^ The Box-Cox method was favored for the primary results. For robustness, we performed additional analyses for each biomarker and each group comparison by estimating pooled effect sizes when only mean-based studies were analyzed, and when only median-based studies were analyzed. Results of all analyses are available in eTable 3.

### Data Availability

The data that support the findings of this study are available from the corresponding author on reasonable request.

## Results

### Study Selection and Characteristics

The search strategy identified 1,186 studies, the majority from Web of Science (n = 719) with the remainder from MEDLINE (n = 467) (eFigure 1). Covidence software automatically identified and removed 279 duplicate studies. The remaining 907 studies underwent title and abstract screening, culminating in 75 studies proceeding to full-text review. Rejection of 34 studies occurred on identification of exclusion criteria, most often (n = 14) due to the AD group not having a clinical diagnosis (e.g., comprising CU individuals with a positive Aβ status). Of the 41 remaining eligible studies, 10 shared an AD cohort. In each case, the study with the larger sample size was retained, resulting in exclusion of 5 further studies (see eTable 1 for details).

Thirty-six studies were included in the meta-analysis, comprising 3,366 AD continuum patients among 54 cohorts (MCI-AD n = 16, AD dementia n = 31, AD dementia + MCI-AD n = 7), and 4,115 CU control participants among 39 cohorts (eTable 4). Two studies measured more than 1 astrocyte biomarker of interest. S100B levels were available in 4 studies (4 cohorts), YKL-40 levels were reported in 11 studies (17 cohorts), and GFAP levels were available in 25 studies (37 cohorts). AUC data were only available for studies reporting GFAP levels (5 studies, 8 cohorts).

Data for all 4 studies measuring S100B, and 8 of the 11 studies measuring YKL-40, were presented as means and SD or SEM. The 3 median-based YKL-40 studies^12-14^ included data for 624 AD continuum patients: over half (50.36%) of the population eligible for meta-analysis. Ten of the 25 studies measuring GFAP levels were reported as medians and IQR or range.^3,13,15-22^

Cumulatively, these 10 studies included 1,135 AD continuum patients, again accounting for approximately half (46.80%) of the total AD continuum population among whom GFAP levels were available for meta-analysis. No study was assessed to have a high risk of bias (eTable 2b).

### Data Transformation

Both transformation techniques previously described yielded similar estimated means and SDs, with the overall effect size of the meta-analyzed results for each biomarker, for all group comparisons, varying by 0.07 at most (eTable 3). Exclusion of median-based studies did not significantly alter the findings, with a maximum difference in effect size of 0.13 when compared with the Box-Cox method, and 0.18 when compared with the second method.

### S100B Analysis

**Figure 1:**
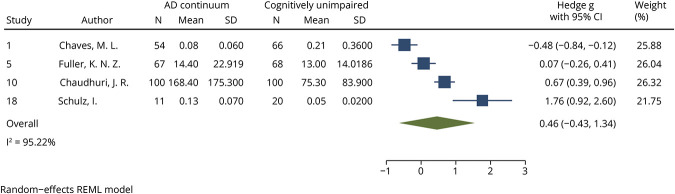
Forest Plot of Studies Comparing Blood S100B Levels Between AD Continuum Patients and CU See eTable 4 for list of studies. AD = Alzheimer disease; AD continuum = cohorts comprising MCI-AD, AD dementia, or a combination of both; CU = cognitively unimpaired; REML = restricted mean likelihood.

S100B levels were meta-analyzed among 232 AD continuum patients (3 cohorts AD dementia n = 165; 1 cohort AD dementia + MCI-AD n = 67) and 254 CU individuals (4 cohorts). There was no significant difference in S100B levels between AD continuum patients and CU individuals (*p* = 0.3118, effect size 0.46, 95% CI −0.43 to 1.34; Figure 1). The *I*^2^ index was 95.22%. No publication bias was detected by visual inspection of a funnel plot (eFigure 2) or by the Egger test (*p* = 0.0935).

### YKL-40 Analysis

Although 11 studies measuring YKL-40 levels met inclusion criteria, data from one of these studies were a significant outlier and were not included in the meta-analysis.^23^ Specifically, the study reported a SD for the AD and CU group's YKL-40 levels 2 orders of magnitude smaller than expected based on all other studies. The 10 meta-analyzed studies included 1,073 AD continuum patients (4 cohorts MCI-AD n = 170, 12 cohorts AD dementia n = 903) and 1,444 CU individuals (11 cohorts).

**Figure 2:**
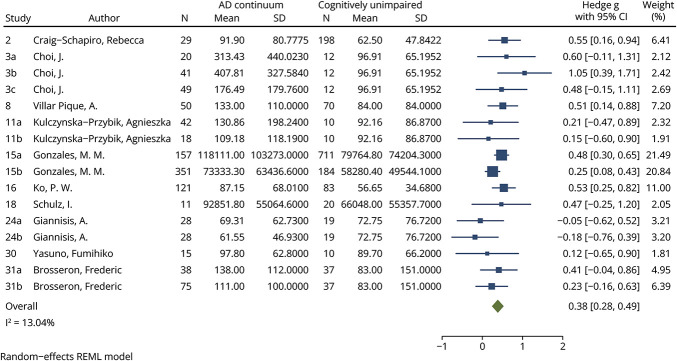
Forest Plot of Studies Comparing Blood YKL-40 Levels Between AD Continuum Patients and CU See eTable 4 for list of studies. AD = Alzheimer disease; AD continuum = cohorts comprising MCI-AD, AD dementia, or a combination of both; CU = cognitively unimpaired; REML = restricted mean likelihood; YKL-40 = chitinase-3-like protein 1.

**Figure 3:**
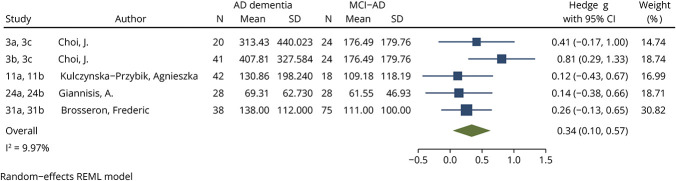
Forest Plot of Studies Comparing Blood YKL-40 Levels Between Patients With AD Dementia and MCI-AD See eTable 4 for list of studies. AD = Alzheimer disease; CU = cognitively unimpaired; MCI-AD = mild cognitive impairment due to Alzheimer disease; REML = restricted mean likelihood; YKL-40 = chitinase-3-like protein 1.

Compared with CU individuals, YKL-40 levels were significantly increased among patients on the AD continuum, including when meta-analyzed based on diagnosis on the clinical spectrum. When YKL-40 levels among AD continuum patients were compared with CU individuals, an effect size of 0.38 (95% CI 0.28–0.49, *p* < 0.0001, Figure 2) was identified. This small effect size was seen when comparing AD dementia patients with CU (effect size 0.43, 95% CI 0.31–0.54, *p* < 0.0001, eFigure 3) and MCI-AD patients with CU (effect size 0.24, 95% CI 0.02–0.46, *p* = 0.033, eFigure 4). Within the AD continuum, YKL-40 levels were significantly higher in patients with AD dementia compared with MCI-AD, with a small effect size (0.34, 95% CI 0.10–0.57, *p* = 0.0048, Figure 3). For all YKL-40 meta-analyses, the *I*^2^ index was below 25%. No significant publication bias was identified using funnel plots (eFigures 5, a–d) or the Egger test for any YKL-40 meta-analysis (AD continuum vs CU *p* = 0.6986, AD dementia vs CU *p* = 0.5635, MCI-AD vs CU *p* = 0.8294, AD dementia vs MCI-AD *p* = 0.8007).

### GFAP Analysis

GFAP levels were meta-analyzed among 2,425 patients on the AD continuum (12 cohorts MCI-AD n = 404, 19 cohorts AD dementia n = 1,568, 6 cohorts AD dementia + MCI-AD n = 453) and 3,168 CU individuals (27 cohorts).

**Figure 4:**
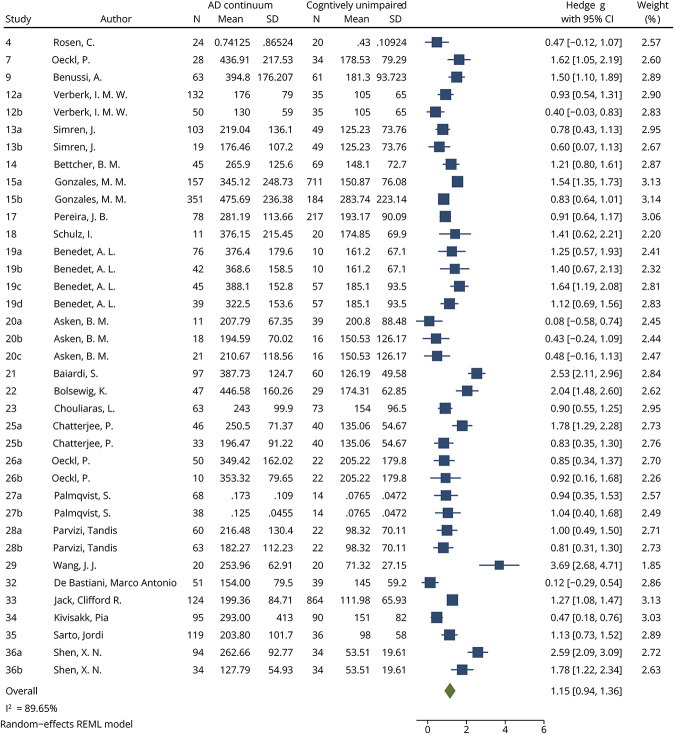
Forest Plot of Studies Comparing Blood GFAP Levels Between AD Continuum Patients and CU See eTable 4 for list of studies. AD = Alzheimer disease; AD continuum = cohorts comprising MCI-AD, AD dementia, or a combination of both; CU = cognitively unimpaired; GFAP = glial fibrillary acidic protein; REML = restricted mean likelihood.

**Figure 5:**
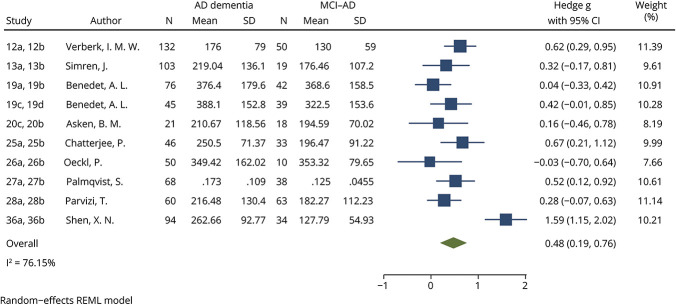
Forest Plot of Studies Comparing Blood GFAP Levels Between Patients With AD Dementia and MCI-AD See eTable 4 for list of studies. AD = Alzheimer disease; CU = cognitively unimpaired; GFAP = glial fibrillary acidic protein; MCI-AD = mild cognitive impairment due to Alzheimer disease; REML = restricted mean likelihood.

Compared with CU individuals, GFAP levels were significantly increased in patients on the AD continuum, with large effect sizes for all comparisons. An effect size of 1.15 (95% CI 0.94–1.36, *p* < 0.0001, Figure 4) was found between AD continuum patients and CU individuals. When meta-analyzed based on clinical diagnosis on the AD clinical spectrum, both MCI-AD and AD dementia patients had significantly increased GFAP levels compared with CU individuals (MCI-AD vs CU effect size 1.02, 95% CI 0.68–1.36, *p* < 0.0001, eFigure 6; AD dementia vs CU effect size 1.33, 95% CI 1.01–1.65, *p* < 0.0001, eFigure 7). GFAP levels were significantly higher in patients with AD dementia compared with MCI-AD, with a small effect size (0.48, 95% CI 0.19–0.76, *p* = 0.0009, Figure 5). Finally, meta-analysis of GFAP AUCs comparing AD continuum patients with CU individuals identified a large and statistically significant mean pooled AUC (0.84, 95% CI 0.77–0.92, *p* < 0.0001, eFigure 8). In all analyses, the *I*^2^ index was more than 75%. Although publication bias was not suspected based on visual inspection of the funnel plot for the AD dementia vs CU comparison (eFigure 9b), the Egger test was significant (*p* = 0.0392). No significant publication bias was identified using funnel plots (eFigure 9, a, c, and d) or the Egger test for any other GFAP meta-analysis (AD continuum vs CU *p* = 0.1835, MCI-AD vs CU *p* = 0.9817, AD dementia vs MCI-AD *p* = 0.4335).

**Figure 6:**
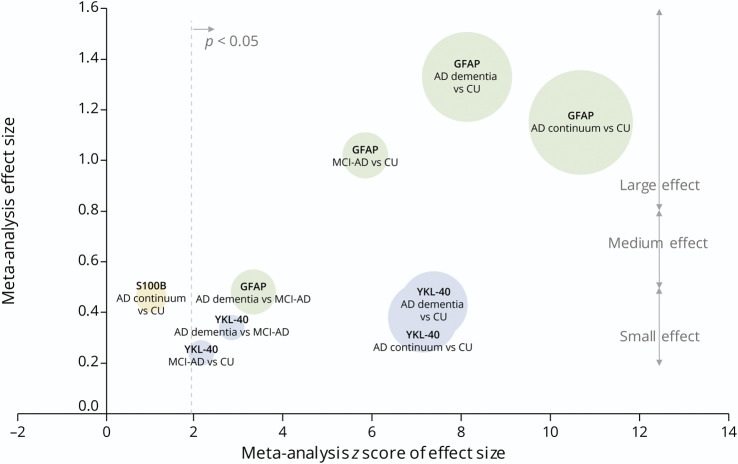
Bubble Plot: Results of All Meta-analyses of Blood Astrocyte Biomarkers Across the AD Continuum The effect size of each meta-analysis is plotted against the *z* score. A *z* score of 1.96 is equivalent to *p* = 0.05. Circle size reflects the total number of participants in the meta-analysis. Raw data are available in eTable 5. AD = Alzheimer disease; AD continuum = cohorts comprising MCI-AD, AD dementia, or a combination of both; CU = cognitively unimpaired; GFAP = glial fibrillary acidic protein; MCI-AD = mild cognitive impairment due to Alzheimer disease.

**Figure 7:**
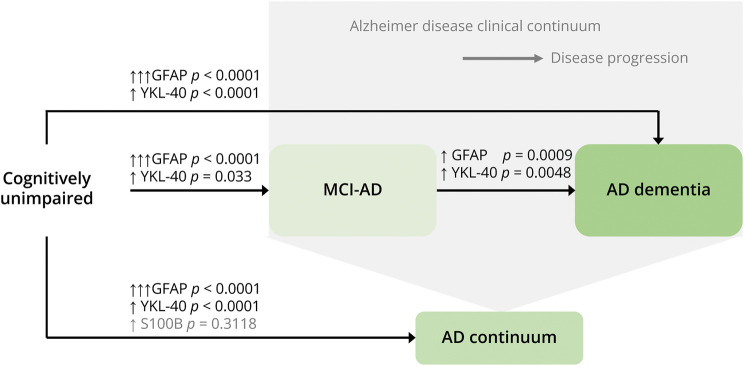
Results From All Meta-analyses Superimposed on a Schematic Representation of the AD Clinical Continuum ↑ = increased biomarker level (small Hedge *g* effect size); ↑↑ = increased biomarker level (moderate Hedge *g* effect size); ↑↑↑ = increased biomarker level (large Hedge *g* effect size); AD = Alzheimer disease; AD continuum = cohorts comprising MCI-AD, AD dementia, or a combination of both; GFAP = glial fibrillary acidic protein; MCI-AD = mild cognitive impairment due to Alzheimer disease.

Figures 6 and 7 summarize the key results from all meta-analyses.

## Discussion

This is the largest meta-analysis of blood astrocyte biomarkers in AD to date. Our findings reveal that in patients across the AD clinical spectrum, blood GFAP and YKL-40 levels are significantly elevated compared with CU individuals, with GFAP demonstrating the largest effect sizes. In addition, both biomarkers show significant elevation within the AD continuum, with higher levels seen in more advanced disease. We did not detect significant differences in S100B levels between CU individuals and those on the AD continuum. Our data suggest that elevations in blood GFAP and YKL-40 reflect AD-related pathology, making them strong candidates for inclusion in AD biomarker panels.

Astrocyte activation is an early event in the AD pathologic cascade, possibly even preceding Aβ plaque formation.^24,25^ As Aβ accrues, astrocyte activity becomes a critical determinant of clinical outcome. Indeed, differences in astrocyte activity may account for why a substantial proportion of Aβ-positive CU individuals do not accrue tau pathology and remain clinically asymptomatic.^26^ Astrogliosis has been pinpointed as a process through which Aβ's neurotoxicity is unleashed: a pathologic bridge linking amyloidosis to downstream tau pathology and AD-related neurodegeneration.^27^ In one study of more than 1,000 CU Aβ-positive individuals, plasma p-tau was only elevated in those who were “positive” for astrocyte reactivity, defined by an elevated plasma GFAP.^27^ Dynamic longitudinal biomarker studies show that plasma GFAP rises sharply in CU individuals on becoming CSF Aβ positive, followed by YKL-40's release into the CSF, before derangements in tau biomarkers begin.^28,29^ This biomarker cascade suggests that early GFAP upregulation mediates the balance between soluble and insoluble Aβ aggregation, with the ensuing YKL-40 signature being linked to Aβ-induced tau phosphorylation.^29,30^ Neuropathologic studies further implicate reactive astrocytes in Aβ plaque evolution. For instance, early diffuse Aβ deposits abound with S100B-overexpressing astrocytes, before dystrophic neurite formation in these deposits.^31^ This finding suggests that S100B, known to be a potent neurite extension factor, is an important mediator of Aβ plaque formation.^32^

The corollary of these observations is that astrogliosis occurs upstream of tau pathology and is a prerequisite to its development. In vitro evidence shows that astrocyte activation exacerbates Aβ-induced neuronal death and is essential to Aβ-driven tau phosphorylation.^33^ Furthermore, reactive astrocytes are pivotal mediators in the formation and evolution of Aβ plaques.

Astrogliosis continues to drive AD pathology across the disease continuum. In the largest AD proteomics study to date, the protein coexpression network most strongly associated with AD was enriched in proteins linked to astrocytes, microglia, and AD genetic risk.^34^ Increased network expression began in preclinical AD, remained elevated into the clinical AD stage, and correlated with cognitive impairment, leading the authors to conclude that glial pathology is a likely causal driver of AD pathogenesis. Given the intimate relationship between astrogliosis and multiple steps in the AD pathologic cascade, astrocyte biomarkers are well-positioned to offer rich insights into the neuropathologic state of a person with AD.

Our meta-analysis identified significant elevations in blood GFAP levels in patients across the AD clinical spectrum compared with CU individuals. All comparisons yielded large effect sizes, including meta-analysis of GFAP AUCs for discrimination of AD continuum patients compared with CU individuals. We also identified differences within the AD continuum, with AD dementia patients having significantly higher GFAP levels than MCI-AD patients, suggesting that GFAP levels increase with clinical disease progression. In longitudinal cohorts comprising healthy adults or those with MCI, the gradient of blood GFAP elevation is greater in those who evolve to dementia.^35,36^ Among CU adults, a higher baseline blood GFAP is associated with the development of clinical AD and more rapid decline in hippocampal volume.^37^ Taken together, our findings support the notion that GFAP elevation is an early and inexorably progressive event in AD pathology, with the gradient and tempo of escalation providing useful clinical information.

Akin to our findings for GFAP, meta-analyzed blood YKL-40 levels were also significantly elevated across the AD clinical spectrum compared with CU individuals. A correlation between biomarker elevation and disease progression along the AD continuum was again identified, with AD dementia patients having significantly higher YKL-40 levels than MCI-AD patients. However, effect sizes were small for all comparisons. A recent neuropathologic study of YKL-40 expression in human brain tissue identified a subset of astrocytes as the source of YKL-40 in AD.^38^ The same study identified a positive correlation between YKL-40 and tau immunoreactivity: a colocalization that implicates the YKL-40 in the AD pathologic cascade. In a murine AD model, deletion of the YKL-40-encoding gene *CHI3L1* decreased amyloid plaque burden, suggesting that YKL-40 promotes Aβ accumulation.^39^ Indeed, a variant in the human *CHI3L1* gene that results in decreased CSF YKL-40 expression is associated with slower AD progression.^39^ Our findings support blood YKL-40 elevation as part of the AD-specific pathologic cascade in both early and more clinically advanced disease.

Activated astrocytes in the AD brain markedly overexpress S100B, evidenced by the grossly elevated tissue levels identified in neuropathologic studies.^40^ When secreted by astrocytes, S100B can exert both trophic and toxic effects on neurons depending on its concentration. At physiologic nanomolar concentrations, S100B stimulates neurite outgrowth^41^ and exerts neuroprotective effects by preventing cell death and loss of mitochondrial function stemming from glucose deprivation.^42^ Conversely, at the pathologically elevated concentrations typical of the AD brain, S100B promotes AD pathology by processes including upregulation of the Aβ precursor protein^43^ and induction of apoptosis.^44^

Despite the demonstrable S100B elevation in the AD brain, our meta-analysis did not identify significant differences in blood S100B levels between CU individuals and AD continuum patients. Several factors likely account for this finding, besides the small number of studies available for meta-analysis. First, although S100B is abundant in astrocytes, it is also produced by a plethora of other glial cells in the CNS, making its serum elevation a nonspecific biomarker of neural damage. Second, S100B is widely expressed by cells outside the nervous system. Finally, anthropomorphic and metabolic factors including elevated BMI,^45^ insulin resistance,^46^ and hypertension^47^ are associated with elevations in serum S100B levels. Ultimately, S100B's nonspecificity to astrocytes or neural tissue, and the readiness with which it is released into the blood in the setting of prevalent medical comorbidities, makes it less likely to be a useful addition to the AD biomarker arsenal.

Reliability is an important consideration when evaluating biomarkers of astrocyte reactivity in AD. A limitation of astrocyte biomarkers is a lack of specificity to any given CNS pathology. For instance, elevations in all of the biomarkers studied in this meta-analysis have been identified in demyelinating diseases, traumatic brain injury, and CNS malignancy. However, recent clinical evidence supports the existence of distinct astrocyte biomarker signatures triggered by brain Aβ and tau pathology.^30^ In a murine model of AD, Aβ and tau induced overlapping reactive astrocyte profiles associated with both deleterious and neuroprotective signals.^48^ Further characterizing the context-specific astrocyte biomarker signature of AD is a priority to enhance our understanding of astrocytes' role in AD pathophysiology. Our findings suggest that elevations in blood GFAP and YKL-40 levels are key components of the AD-specific reactive astrocyte signature, with significantly higher levels correlating with clinical disease progression. Statistically significant results were obtained for all blood GFAP and YKL-40 meta-analyses, further supporting their reliability as biomarkers of astrocyte reactivity in AD.

The results of our meta-analysis have important implications in research and clinical settings. With further validation and clinical implementation, the measurement of both core diagnostic and noncore AD biomarkers (such as those representing astrocyte reactivity) would allow clinicians to generate a patient's individual biomarker profile: a comprehensive characterization of a person's neuropathophysiological state. In a research setting, it may emerge that certain biomarker profiles are associated with different comorbidities, rates of disease progression, or responses to novel therapies, for example.^49^ Translated to clinical practice, the nuanced phenotyping provided by a patient's biomarker profile could enhance a clinician's accuracy when prognosticating, or influence their treatment suggestions based on anticipated drug efficacy. Our findings support blood GFAP and YKL-40 as informative additions to such biomarker profiles for patients with AD. Further studies exploring the associations between biomarker profiles and clinically relevant variables (e.g., rate of clinical decline and pattern of impairment in cognitive domains) are warranted. The therapeutic manipulation of astrocyte activation offers opportunities for drug development, with further studies required to determine the disease-modifying potential of such an approach.^50^

Our study has several strengths. First, we restricted the analysis to studies that directly measured the levels of blood astrocyte biomarkers, rather than those reporting levels of autoantibodies to them, or measurements from plasma astrocyte-derived exosomes. As such, we removed the heterogeneity possibly introduced by a combined meta-analysis of these different species. This may partially explain why our findings differ from those of a previous meta-analysis^4^ which did not find a significant difference in blood GFAP levels between AD and CU populations, based on meta-analysis of 3 anti-GFAP autoantibody studies, 1 study reporting on GFAP levels from astrocyte-derived exosomes, and only 1 study that directly reported blood GFAP levels. Second, we performed comparative meta-analyses across the AD continuum, comparing MCI-AD with AD dementia, as well as CU with both groups separately and combined. Doing so allowed us to demonstrate that both GFAP and YKL-40 levels are not only elevated in patients on the AD continuum compared with CU, but also within the AD clinical spectrum, with higher levels seen in more advanced clinical stages. Third, by not excluding studies that reported biomarker levels as median-based summary statistics, we approximately doubled the AD population sample available for meta-analysis. No significant differences in overall effect size were identified for any biomarker, for any group comparison, when meta-analyses were performed including only mean-based studies (as is typical for meta-analyses), or when median-based studies were added using 2 different methods of data transformation. Our findings suggest that simple data transformation techniques provide a robust method to maximize the number of studies available for inclusion in meta-analyses by facilitating the inclusion of data sets expressed using median-based summary statistics. Excluding otherwise eligible studies simply due to their method of data presentation needlessly dilutes the power of the meta-analysis.

Our study has some limitations. First, the nonsignificant findings from the S100B meta-analysis should be interpreted with caution, given the small sample size available for analysis. Second, we included studies that relied on clinical diagnostic criteria to define their AD population. Given the inherent limitations of a clinical diagnosis, a proportion of patients in the meta-analyzed AD cohorts will represent “false positive” diagnoses without AD pathology. Although the shift to a more objective biomarker-based diagnosis is underway, current practice still relies on using clinical criteria to define AD. As such, we too elected to use a clinical definition of AD to reflect real-world practice and maximize the immediate clinical relevance of our findings. We minimized the effect of this limitation by only including studies that defined their AD population using well-established consensus clinical criteria. Third, the generalization of our findings to the culturally and linguistically diverse population of patients with dementia globally would be strengthened by future studies with greater representation of such participants. Finally, high heterogeneity based on an *I*^2^ index greater than 75% was identified for the S100B meta-analysis, and all GFAP meta-analyses indicate that at least 75% of the observed variance would remain if the sampling error were to be eliminated.^7^ This finding may be due to differences in analyte detection techniques, cohort characteristics, or other factors.

Our findings reveal that blood GFAP and YKL-40 levels are significantly elevated in patients on AD clinical continuum compared with CU individuals. Differences were also identified within the AD clinical spectrum, with significant elevation seen in more advanced disease. Conversely, we did not find a difference in blood S100B levels, possibly due to its lack of specificity to brain tissue, and numerous physiologic factors that confound its interpretation. Our findings suggest that the blood levels of GFAP and YKL-40 reflect AD-related pathology, making both biomarkers strong candidates for inclusion in AD biomarker profiles characterizing a patient's neuropathophysiological state.

## Appendix Authors

**Table:**

Name	Location	Contribution
Sarah Holper, MBBS(Hons)	Population Health and Immunity Division, The Walter and Eliza Hall Institute of Medical Research; Department of Medicine, The Royal Melbourne Hospital, University of Melbourne, Parkville, Australia	Drafting/revision of the manuscript for content, including medical writing for content; major role in the acquisition of data; study concept or design; analysis or interpretation of data
Paula Loveland, MBBS	Population Health and Immunity Division, The Walter and Eliza Hall Institute of Medical Research; Department of Medicine, The Royal Melbourne Hospital, University of Melbourne, Parkville, Australia	Drafting/revision of the manuscript for content, including medical writing for content; major role in the acquisition of data; study concept or design
Leonid Churilov, PhD	Department of Medicine, The Royal Melbourne Hospital, University of Melbourne, Parkville, Australia	Drafting/revision of the manuscript for content, including medical writing for content; study concept or design; analysis or interpretation of data
Dominic Italiano, MBiostat	Department of Medicine, The Royal Melbourne Hospital, University of Melbourne, Parkville, Australia	Drafting/revision of the manuscript for content, including medical writing for content; analysis or interpretation of data
Rosie Watson, PhD	Population Health and Immunity Division, The Walter and Eliza Hall Institute of Medical Research; Department of Medicine, The Royal Melbourne Hospital, University of Melbourne, Parkville, Australia	Drafting/revision of the manuscript for content, including medical writing for content; study concept or design; analysis or interpretation of data
Nawaf Yassi, PhD	Population Health and Immunity Division, The Walter and Eliza Hall Institute of Medical Research; Department of Medicine, The Royal Melbourne Hospital, and Department of Neurology, Melbourne Brain Centre at The Royal Melbourne Hospital, University of Melbourne, Parkville, Australia	Drafting/revision of the manuscript for content, including medical writing for content; study concept or design; analysis or interpretation of data

## Glossary

Aβ: β-amyloid
AD: Alzheimer disease
ACU: area under the receiver operating curve
CU: cognitively unimpaired
GFAP: glial fibrillary acidic protein
IQR: interquartile range
MCI-AD: mild cognitive impairment due to AD
NIA-AA: National Institute on Aging-Alzheimer's Association
YKL-40: chitinase-3-like protein 1
